# Dr. L. W. Cornwell

**Published:** 1906-12

**Authors:** 


Dr. L. W. Cornwell, of Alden, N. Y., died at his home in that village October 22, 1906, at an advanced age. He was a graduate of Castleton (Vt.) Medical College and had been in practice nearly fifty years. He is survived by his wife and by a nephew, Dr. B. W. Cornwell, of Williamsville.

